# Jujuboside A Protects H9C2 Cells from Isoproterenol-Induced Injury via Activating PI3K/Akt/mTOR Signaling Pathway

**DOI:** 10.1155/2016/9593716

**Published:** 2016-05-16

**Authors:** Dandan Han, Changrong Wan, Fenghua Liu, Xiaolong Xu, Linshu Jiang, Jianqin Xu

**Affiliations:** ^1^CAU-BUA TCVM Teaching and Researching Team, College of Veterinary Medicine, China Agricultural University (CAU), Beijing 100193, China; ^2^Beijing Key Laboratory of Dairy Cow Nutrition, College of Animal Science and Technology, Beijing University of Agriculture (BUA), Beijing 102206, China; ^3^Beijing Institute of Traditional Chinese Medicine, Beijing 100010, China

## Abstract

Jujuboside A is a kind of the saponins isolated from the seeds of* Ziziphus jujuba*, which possesses multiple biological effects, such as antianxiety, antioxidant, and anti-inflammatory effects; however, its mediatory effect on isoproterenol-stimulated cardiomyocytes has not been investigated yet. In this study, we tried to detect the protective effect and potential mechanism of JUA on ISO-induced cardiomyocytes injury. H9C2 cells were treated with ISO to induce cell damage. Cells were pretreated with JUA to investigate the effects on the cell viability, morphological changes, light chain 3 conversion, and the activation of PI3K/Akt/mTOR signaling pathway. Results showed that ISO significantly inhibited the cell viability in a time- and dose-dependent manner. JUA pretreatment could reverse the reduction of cell viability and better the injury of H9C2 cells induced by ISO. Western blot analysis showed that JUA could accelerate the phosphorylation of PI3K, Akt, and mTOR. Results also indicated that JUA could significantly decrease the ratio of microtubule-associated protein LC3-II/I in H9C2 cells. Taken together, our research showed that JUA could notably reduce the damage cause by ISO via promoting the phosphorylation of PI3K, Akt, and mTOR and inhibiting LC3 conversion, which may be a potential choice for the treatment of heart diseases.

## 1. Introduction

Activation of *β*-adrenoceptors (*β*-AR) is a main reason for myocardial injury [1]. Isoproterenol (ISO), a synthetic catecholamine and the *β*-AR agonist used to treat bradycardia or heart block, could produce severe myocardial stress and infarct like necrosis with decreased myocardial compliance and inhibition of diastolic and systolic function when administered in supramaximal dose [2–4]. It has been reported that isoproterenol produced free radicals and stimulated lipid peroxidation, which is one of the causative factors for irreversible damage to the myocardial membrane [3, 5, 6]. It is also documented that the metabolic, morphological, and pathophysiological alterations are induced by ISO in experimental animals [7, 8]. Therefore, ISO-induced changes can be used as a model for studying the effect of protective agents on the processing of myocardial injury.

Semen ziziphi spinosae (SZS), the mature seed of* Ziziphus jujuba *Mill. var.* spinosa *(Bunge) Hu ex H. F. Chow, has been used as a sedative medicine in China, Japan, Korea, and other oriental countries for over one thousand years [9, 10]. Jujuboside A (JUA), the triterpene saponins isolated from SZS, has been proved to be a major active component of SZS [11, 12]. Previous studies showed that JUA can exert antioxidant, antianxiety, anti-inflammatory, and hypnotic-sedative activities and reduce the cell apoptosis [13–15]. However, there is no investigation focusing on the protective effect of JUA on ISO-induced myocardial injury.

In this study, ISO-induced H9C2 cell damage was used as an experimental model. We performed a preliminary assessment on cytotoxicity of JUA in cardiomyocytes and investigated the protective effect of JUA against ISO-induced injury of H9C2 cell.

## 2. Materials and Methods

### 2.1. Reagents and Antibodies

JUA [>98% high-performance liquid chromatography (HPLC) purity] was purchased from National Institutes for Food and Drug Control (Beijing, China). ISO and 3-(4,5-dimethylthiazol-2-yl)-2,5-diphenyltetrazolium bromide (MTT) were purchased from Sigma-Aldrich Chemical (Sigma, USA). Dulbecco's modified Eagle medium (DMEM) high glucose, fetal bovine serum (FBS), and antibiotic-antimycotic were purchased from Gibco (Grand Island, NY, USA). Bicinchoninic acid (BCA) protein assay kit was purchased from Pierce (Rockford, IL, USA). Antibodies for *β*-actin (#8457), PI3K (#4292), p-PI3K (#4228), Akt (#4685), p-Akt (#4060), mTOR (#2972), p-mTOR (#5536), and LC3 (#12741) were purchased from Cell Signaling Technology (Danvers, MA, USA). The goat anti-mouse antibody was purchased from Li-cdr Odyssye® (Lincoln, NE, USA).

### 2.2. Cell Culture and Treatment

H9C2 cells were purchased from the Cell Resource Center of Chinese Academy of Medical Sciences (Peking Union Medical College, China). Cells were cultured in DMEM supplemented with 10% FBS and antibiotics (100 U/mL penicillin and 100 U/mL streptomycin) at 37°C in a humidified atmosphere of 5% CO_2_ and subcultured in 0.05% trypsin. The medium was replenished every 1~2 days. JUA and ISO were diluted in DMEM (1 mg/mL). All cells were washed with phosphate buffer saline (PBS) and serum-starved for 2 h before incubation with JUA or ISO. JUA was dissolved in dimethylsulfoxide (DMSO), and the final DMSO concentration was ≤0.05% (v/v).

### 2.3. Cell Viability Assay

Cell viability was determined by MTT reduction assay. In brief, H9C2 cells were preincubated with DMEM containing 10% FBS overnight in 96-well plates at a density of 5 × 10^4^ cells per well. After cells were grown to 90% confluence, all cells were washed twice with PBS and serum-starved for 2 h. According to the experimental design, the culture medium was replaced (with or without ISO or the other compounds) and then cultured for 6 h. The mediums then were removed, and solution containing 10% MTT and DMEM was added to each well. The cells were incubated at 37°C for 4 hours, the supernatants were removed, and the formazan crystals were dissolved in 150 *μ*L DMSO (Sigma, USA). Absorbance was recorded at a wavelength of 490 nm and reference wavelength of 630 nm using a microplate reader (BIO-RAD, Foster, California, USA). The cell viability is expressed as the ratio of the absorbance in control cultures.

### 2.4. Treatment and Morphology Observation

Briefly, cells were treated with JUA or ISO according to the experimental design. Following each specific treatment, cell morphology was observed using a phase-contrast inverted biological microscope (IX71/IX2, Olympus, Japan), scanning electron microscopy (SEM; S-3400N, Hitachi, Tokyo, Japan), and transmission electron microscopy (TEM; 1230, JEOL, Tokyo, Japan). For ultrastructure studies, cardiomyocytes were harvested and fixed with 3.0% glutaraldehyde and 1.5% paraldehyde, washed three times in PBS, and postfixed in cold 1% osmium tetroxide. After dehydration with graded series of alcohol, all samples were freeze-dried, coated in a sputter-coater with a layer of gold embedded in epoxy resin (EPON812, Emicron, Shanghai, China), and examined using a Hitachi S-3400N scanning electron microscope operated at 15 kV. Other ultrathin sections were stained with saturated uranyl acetate in 50% ethanol and lead citrate, and the ultrastructure of the H9C2 cardiomyocytes was examined by TEM.

### 2.5. Western Blot Analysis

H9C2 cells (1 × 10^6^), cultured in tissue culture flasks for 24 h, were pretreated with JUA (5, 10, or 20 *μ*M) 3 h prior to treatment with ISO (100 *μ*M) for 6 h in a 37°C, 5% CO_2_ incubator. Cells were then harvested on ice, washed twice using ice-cold PBS, and suspended in 500 *μ*L lysis buffer supplemented with protease inhibitor. Cells were extracted using a total protein extraction kit (Biochain, Hayward, CA, USA) and quantified using a BCA protein assay kit (Pierce, Rockford, USA) according to the manufacturer's instructions. Proteins were separated by sodium dodecyl sulphate polyacrylamide gel electrophoresis and transferred to nitrocellulose membranes (Pierce, Rockford, USA). Membranes were blocked using SuperBlock T20 (TBS) blocking buffer (#37536, Pierce) for 2.5 h at room temperature and incubated overnight at 4°C with specific primary antibodies. The secondary antibody was at a dilution of 1 : 15000, and the proteins were incubated with the secondary antibody for 1 h. Then, the antigen was visualized and analyzed using the Odyssey Infrared Imaging System (LI-COR Biosciences, Lincoln, NE). Blots were normalized by use of *β*-actin to correct for differences in loading of the proteins because it did not change significantly in the proteome profiles. Quantification of digitized images of western blot bands from three biological replicates was performed using Image J (National Institutes of Health, NY, USA).

### 2.6. Statistical Analysis

Unless otherwise indicated, all data were obtained from at least 3 independent experiments performed in triplicate. Data was expressed as mean and standard error of the mean and assessed by the one-way analysis of variance (ANOVA) followed by Duncan's test for multiple comparisons and post hoc tests using SPSS 19.0 (SPSS, Inc., and IBM Company, Chicago, IL, USA). *P* value of 0.05 or 0.01 was considered statistically significant.

## 3. Results

### 3.1. Effect of JUA on Cell Viability* In Vitro*

**Figure 1:**
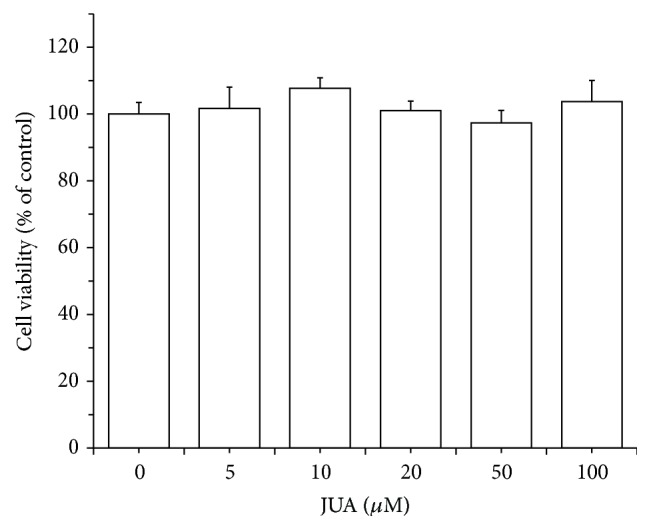
Cytotoxicity of JUA on H9C2 cells. Effect of CGA on the viability of H9C2 cells was measured using MTT assay. Cells were incubated with JUA with various concentrations (5, 10, 20, 50, and 100 *μ*M) of CGA for 24 h. Data represent means ± SEM of six separate experiments and differences between mean values were assessed by one-way ANOVA.

In order to examine the cytotoxic effect of various concentrations (5, 10, 20, 50, and 100 *μ*M) of JUA on H9C2 cells, cell viability was determined using the MTT assay. Results showed that JUA, in concentrations from 0 to 100 *μ*M, had no cytotoxic effect on H9C2 cells (Figure 1).

### 3.2. JUA Improves Cell Viability after ISO Exposure

**Figure 2:**
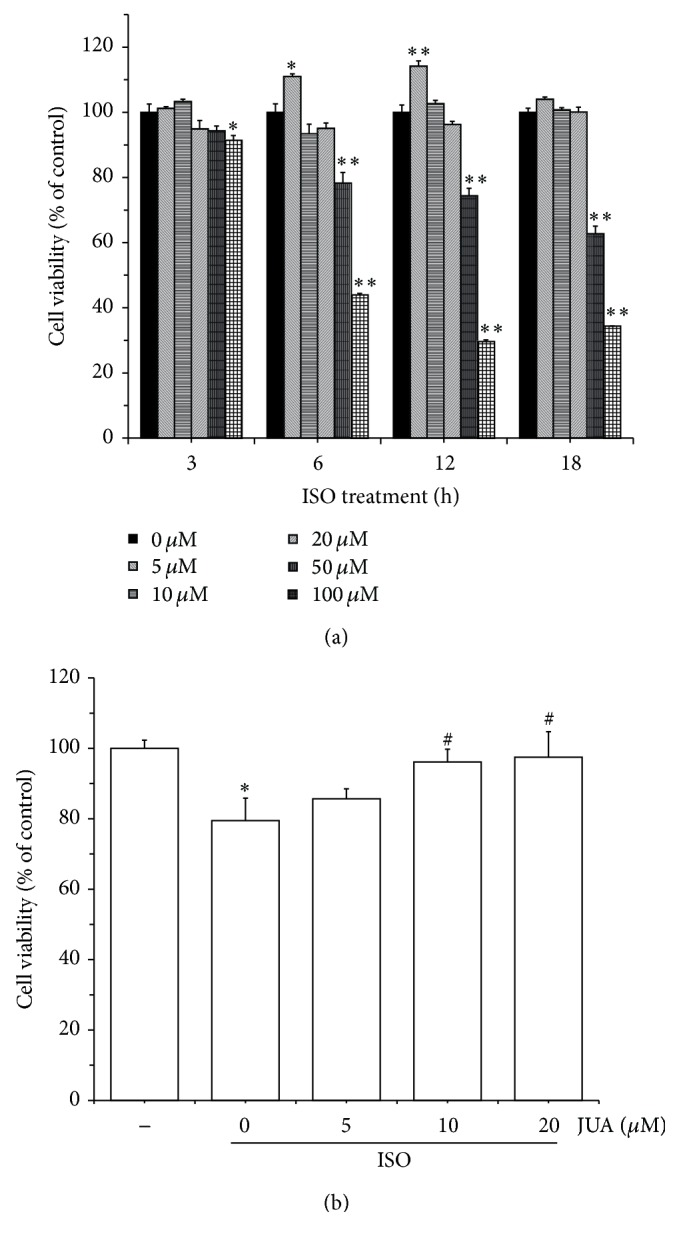
Protective effect of JUA on ISO-induced cytotoxicity in H9C2 cells. (a) Cells were treated with different concentrations of ISO (0~200 *μ*M) for 3, 6, 12, and 18 h. The cell viability was determined by MTT assay. (b) Cells were pretreated with or without JUA at the indicated concentrations for 3 h and then incubated in the presence of ISO (100 *μ*M) for a further 6 h. The cell viability was determined by MTT assay. Data represent means ± SEM of six separate experiments and differences between mean values were assessed by one-way ANOVA. ^*∗*^ *P* < 0.05 and ^*∗∗*^ *P* < 0.01 versus control group; ^#^ *P* < 0.05 versus group treated with ISO only.

To determine the effect of H9C2 cell injury induced by ISO, cell viability was tested by MTT assay after cells were treated with various concentrations of ISO for 3, 6, 12, and 18 h. As shown in Figure 2(a), cell viability was not changed after being treated with 10, 20, 50, and 100 *μ*M of ISO for 3 h. After being stimulated with 200 *μ*M of ISO for 3 h, cell vitality was significantly inhibited (*P* < 0.05). An obvious decrease (*P* < 0.01) in cell viability occurred after treatment with 100 and 200 *μ*M of ISO for 6, 12, and 18 h, and the effect of cell viability inhibition induced by ISO was more serious. According to the results, concentration of 100 *μ*M of ISO for 6 h was chosen to be the model condition of H9C2 damage for further research. To assess the protective effect of JUA on ISO-induced cytotoxicity, the H9C2 cells were treated with JUA (5, 10, and 20 *μ*M) for 3 h, and, then, ISO (0 or 100 *μ*M) was added and incubated for 6 h. Subsequently, cell viability was also detected by MTT assay. The results (Figure 2(b)) demonstrated that JUA significantly enhanced the survival rates of the cells after exposure to ISO, and with increased amounts of JUA, the cell survival rates showed an increasing trend.

### 3.3. JUA Alleviates ISO-Induced Cell Damage in H9C2 Cells

**Figure 3:**
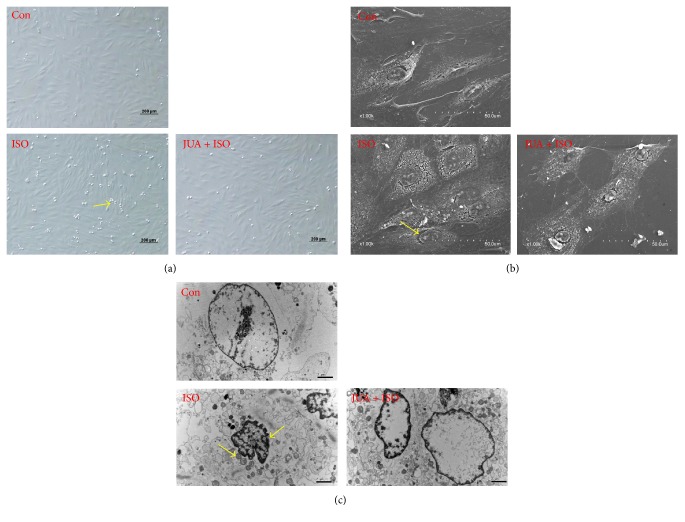
Effect of JUA on ISO-induced morphological changes in H9C2 cells. Cells were pretreated with or without JUA (100 *μ*M) for 3 h and then incubated in the presence of ISO (100 *μ*M) for a further 6 h. Changes in cell morphology were observed using a phase-contrast inverted biological microscope (a), scanning electron microscope (b), and transmission electron microscope (c). Con is the control cells without any medicine processing; ISO is the model cells treated with 100 *μ*M of ISO; JUA + ISO is the medicine cells treated with JUA and ISO.

To evaluate the degree of characteristic morphological changes of H9C2 cells, we monitored cytomorphology using phase-contrast inverted biological microscope, scanning electron microscope, and transmission electron microscope. As shown in Figure 3, treatment with ISO (100 *μ*M) for 6 h induced obvious morphological changes in H9C2 cells. ISO induced pronounced cell damage as displayed by cell shrinkage and gradual detachment from culture dishes (Figure 3(a)). SEM showed normal elongated spindle cells and may help in electrophysiological properties [16], characterized with complete grain, dense, and delicate surface. ISO induced abnormal cellular microstructures characterized with irregular-arranged cell shape; cell surface protein became sparser and increased nuclear gap (Figure 3(b)). Electron microscopy analysis of H9C2 cells revealed obvious alterations in the structure of cardiomyocytes and the cellular architecture. Ultrastructural images of H9C2 cells using TEM showed obvious nuclear chromatin margination, aggregation, and condensation and mitochondrial vacuolization in ISO-treated cells (Figure 3(c)). Pretreatment with JUA (10 *μ*M) for 3 h dramatically alleviated the morphological changes. This finding suggests that JUA produces significant protection in H9C2 cells.

### 3.4. Influence of JUA on ISO-Induced PI3K/Akt Signaling Pathway

**Figure 4:**
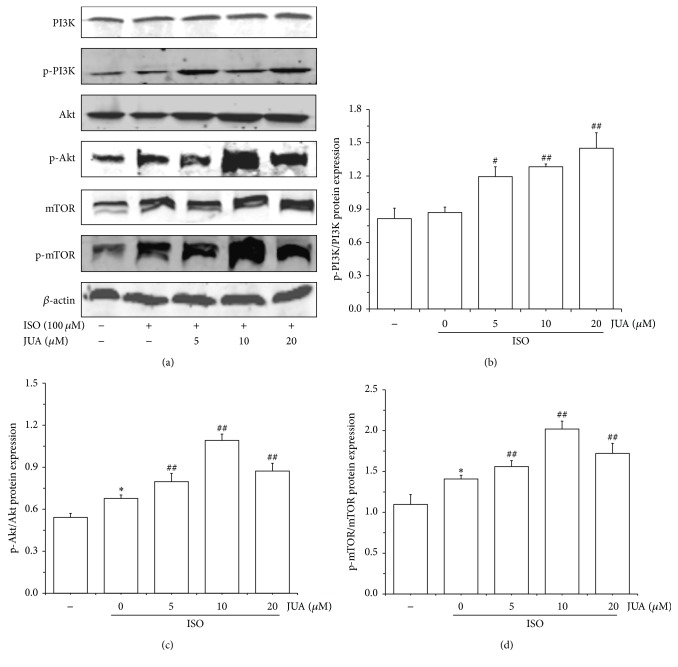
Promotion of ISO-induced PI3K/Akt pathway activation by JUA. Cells were pretreated with various concentrations (5, 10, and 20 *μ*M) of JUA and exposed to 100 *μ*M of ISO for 6 h; proteins samples were prepared at the indicated time points and analyzed by western blotting. (a) The differences of signaling protein level including phospho- and total-PI3K, AKT, and mTOR proteins expression detected with western blotting analysis. ((b), (c), and (d)) Ratio between phosphorylated and total protein levels was calculated and relative protein quantification in treated versus control extracts was calculated by densitometric analysis. Beta-actin was probed as a loading control. Data represent the mean ± SEM of three independent experiments, and differences between mean values were assessed by one-way ANOVA. ^*∗*^ *P* < 0.05 versus control group; ^#^ *P* < 0.05 versus group treated with ISO only; ^##^ *P* < 0.01 versus group treated with ISO only.

To further expound the mechanism of protective effect of JUA on ISO-induced myocardial injury, we then investigated the activation of PI3K/Akt/mTOR signaling pathway after intervention of JUA on ISO-induced H9C2 cells. Cell proteins were extracted for western blotting analysis. We assessed the effect of JUA on ISO-induced phosphorylation of Akt, PI3K, and mTOR using three different phosphospecific antibodies. Results indicated that the expression of phosphorylation of Akt and mTOR was significantly strengthened by ISO (*P* < 0.05), and the phosphorylation of PI3K, Akt, and mTOR levels was all enhanced to some degree in JUA-pretreated cells compared with ISO-treated cells (*P* < 0.01, Figure 4).

### 3.5. Influence of JUA on ISO-Induced LC3 Conversion

**Figure 5:**
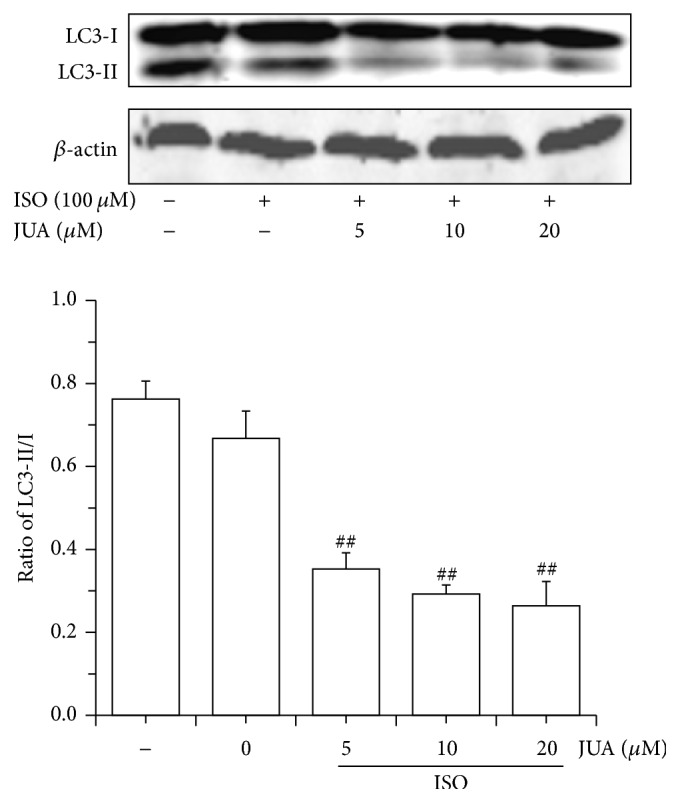
Inhibition of ISO-induced LC3 conversion by JUA. Effect of JUA in different concentrations on ISO-induced LC3-II/I protein level in H9C2 cells. Cells were pretreated with various concentrations (5, 10, and 20 *μ*M) of JUA and exposed to 100 *μ*M of ISO for 6 h; proteins samples were prepared at the indicated time points and analyzed by western blotting. LC3 was analyzed using specific anti-LC3 antibody. Ratio of LC3-II/I protein levels was calculated and relative protein quantification in treated versus control extracts was calculated by densitometric analysis. Beta-actin was probed as a loading control. Data represent the mean ± SEM of three independent experiments, and differences between mean values were assessed by one-way ANOVA. ^##^ *P* < 0.01 versus group treated with ISO only.

LC3 is essential for final autophagosome formation and thus serves as an autophagosome marker. The ratio of LC3-II/I most likely indicated the levels of autophagy. Therefore, western blot analysis was performed to detect LC3-II/I level. Correspondingly, the ratio of LC3-II/I in JUA-pretreated cells decreased remarkably (*P* < 0.01) compared to control group and ISO-stimulated group (Figure 5).

## 4. Discussion

ISO, a *β*-adrenergic agonist and synthetic catecholamine, can be employed at submaximal dose as a noninvasive method to induce myocardial lesions in rodents [17, 18]. Recent studies have been conducted to evaluate myocyte damage following exposure to this drug in rat and monkey species [4]. In this study, H9C2 cells were exposed to ISO to perform a myocardial toxicity model triggered by persistent excitation of *β*1 receptor; we found a significant inhibition of cell viability of H9C2 cells induced by ISO in a time- and dose-dependent manner (Figure 2(b)). In addition, we found that ISO-induced morphology of H9C2 was changed and cell microstructure was destroyed. In model group, morphological changes including fuzzy cell boundary, cellular atrophy, disorderly or missing microvilli, and different degrees of cell collapse were observed by SEM. Subcellular structure changes including nuclear cavitation, swelling or rupture of microvilli, hazy mitochondrial structures, and loose cytoplasmic matrix structure were studied by means of TEM (Figure 3). Therefore, an excessive stress response stimulated by ISO has been confirmed in cardiomyocytes, which will cause damage to cells and reduce cell viability.

JUA, a classic natural product extracted from SZS, has been efficiently used for insomnia relief and considered to be the major effective pharmacological active constituent [13, 19]. In our study, MTT assay showed that JUA did not exhibit cytotoxic effect in concentrations from 0 to 100 *μ*M when treating H9C2 cells (Figure 1). Moreover, JUA effectively reversed the inhibition of cell viability caused by ISO (Figure 2(a)). Recent researches also found that JUA has certain effect on anti-injury effect and neuroprotective and cardioprotective activity via antioxidative and anti-inflammatory effects in dementia animals [11, 20, 21].

The PI3K/AKT/mTOR pathway is one of major signal transduction pathways responsible for regulating cell growth, proliferation, survival, apoptosis, and malignant transformation and is frequently hyperactivated in most cancers [22–24]. The lipid products of phosphoinositide 3-kinase (PI3K) provide localized membrane anchors for the assembly of various signaling proteins, which have domains that bind to D3 phosphorylated phosphoinositides [25, 26]. The well-established downstream target of PI3K is the serine-threonine kinase Akt (also known as protein kinase B, PKB), which is considered to be a key mediator of the PI3K signaling pathway and transmits survival signals from growth factors. It participated in the regulation of cellular signaling networks linked to the survival, growth, proliferation, metabolism, and differences in cell functions and controlled major cell functions through a number of downstream effectors, including p70S6K/p85S6K, 4E-BP1, NF-*κ*B, and mammalian target of rapamycin (mTOR) [27–30]. mTOR is a highly conserved serine-threonine protein kinase that belongs to the PI3K family and plays an important role in signal transduction pathways that control cell proliferation, survival, angiogenesis, and protein translation [31–34]. Therefore, we focus on the role and regulatory mechanisms of PI3K/Akt/mTOR signaling pathway in the process of JUA exhibited proliferation on H9C2 cell. We found that JUA promotes H9C2 cardiomyocytes cell proliferation through strengthening the activation of PI3K/Akt/mTOR signaling pathway.

mTOR played a vital role in regulating cell proliferation, growth, differentiation, and survival, controlling a cell that undergoes programmed cell death type I (apoptosis) or type II (autophagy) [35]. Activated Akt is the downstream effector of PI3K, which can stimulate mTOR to negatively regulate autophagy [36]. Activation of the PI3K-Akt-mTOR and Akt-tuberous sclerosis complex- (TSC-) mTOR pathways inhibits autophagy, whereas the loss of signaling through this cascade removes the negative repression of mTOR. Therefore, there is a direct link between autophagy and the mTOR signaling pathway [37]. Microtubule-associated protein light chain 3 (LC3), a mammalian homologue of yeast Apg8p, was used as a marker of autophagy induction, because cytosolic LC3-I is processed to its lipidated LC3-II form upon autophagy induction [38]. LC3 is initially synthesized in an unprocessed form, pro-LC3, which is converted into the proteolytically processed form LC3-I, lacking amino acids from the C-terminus, and is finally modified into a phosphatidylethanolamine-conjugated form LC3-II [39]. LC3-II is the protein marker that is reliably associated with phagosomes, sealed autophagosomes, and mature autophagosomes/lysosomes [40]. Once the autophagosome and lysosome integrated, LC3-II in autophagosome degraded by hydrolase in lysosome. The expression of LC3-II is proportional to the number of autophagic vacuoles, and LC3-II is regarded as a marker of autophagosome formation [41]. That is to say, we can judge the activity of autophagy by detecting the expression of LC3-II or the ratio of LC3-II/LC3-I [42]. Western blot analysis revealed significantly increased levels of p-Akt and p-mTOR and decreased LC3 conversion in H9C2 cells, indicating that the PI3K/Akt/mTOR pathway may be involved in the regulation of autophagy by JUA.

## 5. Conclusions

In conclusion, our investigation indicated that JUA has potential protective effect on ISO-induced damage in H9C2 cells by accelerating the activation of PI3K/Akt/mTOR signaling pathway and decreasing LC3 conversion. These observations therefore suggest that JUA, a saponin from semen ziziphi spinosae, possesses potential anti-injury activity and beneficial characteristics for cardiovascular diseases. However, other possible pathways and targets related to the anti-injury effect of JUA need to be researched in the future.
